# Reducing Physical Risk Factors in Construction Work Through a Participatory Intervention: Protocol for a Mixed-Methods Process Evaluation

**DOI:** 10.2196/resprot.5648

**Published:** 2016-05-26

**Authors:** Jeppe Ajslev, Mikkel Brandt, Jeppe Lykke Møller, Sebastian Skals, Jonas Vinstrup, Markus Due Jakobsen, Emil Sundstrup, Pascal Madeleine, Lars Louis Andersen

**Affiliations:** ^1^ National Research Centre for the Working Environment Copenhagen Denmark; ^2^ Physical Activity and Human Performance group - SMI Dept. of Health Science and Technology Aalborg University Aalborg Denmark; ^3^ Centre for Working Life Research Roskilde University Roskilde Denmark

**Keywords:** musculoskeletal disorders, process evaluation, intervention study, mixed-methods study, social science, physical exposure, masculinity, construction work

## Abstract

**Background:**

Previous research has shown that reducing physical workload among workers in the construction industry is complicated. In order to address this issue, we developed a process evaluation in a formative mixed-methods design, drawing on existing knowledge of the potential barriers for implementation.

**Objective:**

We present the design of a mixed-methods process evaluation of the organizational, social, and subjective practices that play roles in the intervention study, integrating technical measurements to detect excessive physical exertion measured with electromyography and accelerometers, video documentation of working tasks, and a 3-phased workshop program.

**Methods:**

The evaluation is designed in an adapted process evaluation framework, addressing *recruitment*, *reach*, *fidelity*, *satisfaction*, *intervention delivery*, *intervention received*, and *context* of the intervention companies. Observational studies, interviews, and questionnaires among 80 construction workers organized in 20 work gangs, as well as health and safety staff, contribute to the creation of knowledge about these phenomena.

**Results:**

At the time of publication, the process of participant recruitment is underway.

**Conclusions:**

Intervention studies are challenging to conduct and evaluate in the construction industry, often because of narrow time frames and ever-changing contexts. The mixed-methods design presents opportunities for obtaining detailed knowledge of the practices intra-acting with the intervention, while offering the opportunity to customize parts of the intervention.

## Introduction

Musculoskeletal disorders are a major economic, social, and health challenge in the construction industry, as well as in other occupations characterized by high levels of physical exertion at work [1,2]. Physical exertion and strenuous work tasks such as heavy lifting, pushing, dragging, and working in awkward positions are known to increase the risk of developing musculoskeletal pain [3]. Considering that most of these risk factors are a natural part of the everyday practices in construction work, it is surprising that only a few interventions are aimed at reducing the physical exposure associated with construction work. Furthermore, studies of these interventions have reported very low degrees of measurable success [4,5]. In general, interventions targeting the prevention of musculoskeletal disorders have shown very limited effect, or the evidence has been characterized as being of low quality [6-8], except for a few studies focusing on increasing individual workers’ physical capacity and thereby their resilience against physical exposure at work [6,9-11].

As a consequence of this limited success in preventing musculoskeletal disorders in the working population in general, and for construction workers in particular, evaluators and researchers have called for more multidisciplinary-anchored interventions [4,6,8]. Furthermore, to achieve a successful intervention and for sustaining organizational change and engagement, several sources point out that the inclusion of relevant parties (ie, health and safety professionals, workers, and management) is pivotal [12-15].

In response to this, we have tailored an intervention that engages workers and management, and integrates the use of technical quantitative measurements of excessive physical workload measured with electromyography and accelerometers, video documentation of working tasks, and a 3-phased workshop program. Whereas Brandt et al [16] described the protocol for the technical physical measurements and quantitative survey on physical exertion and pain, this study protocol describes the mixed-methods process evaluation of social, subjective, and organizational processes that play important roles in the outcome of the intervention project.

Research shows that reducing physical exertion in construction work is a complex issue [15,17]. As Nielsen et al [18] emphasized, evaluations of organizational research must be contextually grounded. To ensure that we obtain the best possible knowledge from our intervention, we must carefully tailor the process evaluation to identify the agential roles of the various agencies intra-acting with rationalities at work—with, in, and between workers, in the physical characteristics of construction workers and work, and in the organizational practices of companies where the interventions are implemented.

Construction work is a broad category that encompasses many different trades composed of different tasks, skillsets, and cultural norms. However, physically exerting work is a common denominator. Across carpentry, bricklaying, concrete work, scaffolding, plumbing, etc, the entangled body and subjectivity of the worker is a main resource of production, a point that takes a line of arguments to unfold. However, it is highly important for understanding the social framing of the intervention context.

The necessity of undertaking tasks in a physically exerting manner is embedded in the organization of work, where planned availability and usage of technical assistive devices play important roles. Technical assistive devices have the potential to substantially decrease physical exertion at work, but numerous political, organizational, and subjective agencies challenge the development, proliferation, and usage of such technical assistive devices [19-21]. Thus, interventions addressing the increased use of assistive devices should carefully consider this context in the process evaluation.

One of these agencies lies in the material and organizational orchestration of work that takes place at geographically delimited sites for a certain amount of time, after which, on project completion, the whole organization breaks up to be reconfigured at new worksites. Here the tasks involve many of the same skills but are usually still markedly different, and pose new and different flows of processes, still containing physically exerting work tasks. For this reason, planning of construction sites is a complicated matter, in which the usage of technical assistive devices is sometimes not thoroughly considered. This means that adequate measures for reducing physical exertion may not always be present in the construction process. Without a rigorous process evaluation, important opportunities and barriers in this regard could easily be missed.

At a political and societal level, the competition among entrepreneurs to secure projects has been described as a barrier for implementing initiatives prioritizing health and safety [20,22]. In previous studies, entrepreneurs described how health and safety measures are often the target of budget cuts. When bids are produced, time pressure as a result of tight planning schedules is also very likely to affect all phases of production, leading to a faster work pace among workers [22]. This may also reduce the incentive for investment in technical assistive devices, particularly in companies where profit margins are relatively narrow. Consequently, process evaluations of interventions should also consider time pressure and budget constraints.

Furthermore, many construction projects are completed on some form of performance-based payment (eg, piece rate), which provides the worker with an economic incentive for working faster with less variation, and not using technical assistive devices unless they directly increase production [22-24]. This can play an important role in relation to the effectiveness of our intervention. Part of the process evaluation should therefore also evaluate incentives for increased bodily strain in the above-mentioned forms. The options for working for performance-based wages are guaranteed in agreements between unions and employers’ associations, and are therefore tied to political decision-making processes.

In previous studies, communication between the managerial staff and workers has been suggested as a potential barrier to improving health and safety at the workplace [12,25]. Even though there is a dedicated health and safety organization at every major Danish construction site, the workers’ representatives report being ignored by management, and the management report that workers display a lack of interest in both health and safety, and the organizational need for cooperation to obtain smooth production [26]. This may potentially affect experiences of physical exertion and pain among workers, as higher levels of worker influence on health and safety has been linked to lower levels of exertion and pain [27]. This, at times, negative relationship may obstruct implementing and anchoring the intervention within the organization, and is an issue that demands special attention from researchers conducting the intervention to avoid being seen as either employees’ or management’s allies. Thus, evaluation of communication at the workplace is also an important part of the process evaluation.

In addition, many construction workers embody and socially transmit traditional masculine working-class qualities [28,29], such as endurance, strength, self-reliance, pain habituation, and breadwinning, as positive identity characteristics [20]. Being able to display and participate in discursive-material practices, reconfiguring the worker in accordance with these qualities, in many cases functions as a parameter for maintaining social position in the work gang and a job within the company. Not participating in these traditional masculine working-class practices may be costly to the worker, as sickness absence is frowned upon and employment can be terminated with only 1 day’s notice unless the worker has been employed by the company for more than 1 year [22]. As such, the need to participate in these practices can increase physical exertion and may, furthermore, complicate intervention, as taking care of the individual worker’s body is likely a low priority for both colleagues and the company. Thus, the process evaluation should also evaluate how worker identity plays into the intervention.

In our evaluation, all these agencies and their entangled roles are points of attention, as we aim to understand the facilitators and barriers to reducing physical exertion. The purpose of the process evaluation is to investigate how the intervention intra-acts with both the worker’s body and subjectivity, as well as social and organizational relations acting as facilitators and barriers to reducing physical exertion. An additional aim is to perform analyses using the mixed-methods design to evaluate potential consequences of the intervention in terms of productivity, sickness absence, and time frames.

## Methods

### The Intervention

The intervention is taking place in a design containing several phases as thoroughly described by Brandt et al [16].

In the first phase of the intervention, 20 construction gangs (N=80 workers) will be randomly assigned at a cluster level to a participatory intervention group or a control group. We will record in situ physical workload during a working day using technical measurements (electromyography, accelerometers, and video recordings) before and after the intervention. Based on these measurements, a physical load matrix for each worker will be developed. This matrix is based on outcomes obtained from the analyses of the simultaneously recorded electromyograms and accelerometer data.

The second phase is designed as a participatory process consisting of 3 workshops: 1) workshop I at baseline, involving presentation of video clips of the work tasks with excessive physical load customized for each gang, followed by a participatory development of solutions on how to reduce excessive workloads, leading to the development of an action plan on how to implement these solutions at the workplace, 2) workshop II, where the implemented solutions will be further developed and qualitatively evaluated during group discussion, 3) workshop III at follow-up to enhance long-term organizational sustainability of the implemented solutions. All workshops will aim to include researchers, workers, occupational health and safety (OHS) staff at the companies, and a management representative as participants. We will facilitate the elaboration of solutions aimed at lowering the physical exertion related to the participants’ suggestions. The control groups are not targeted in the evaluation study and are therefore not addressed in this protocol.

### Evaluation Design

**Figure 1:**
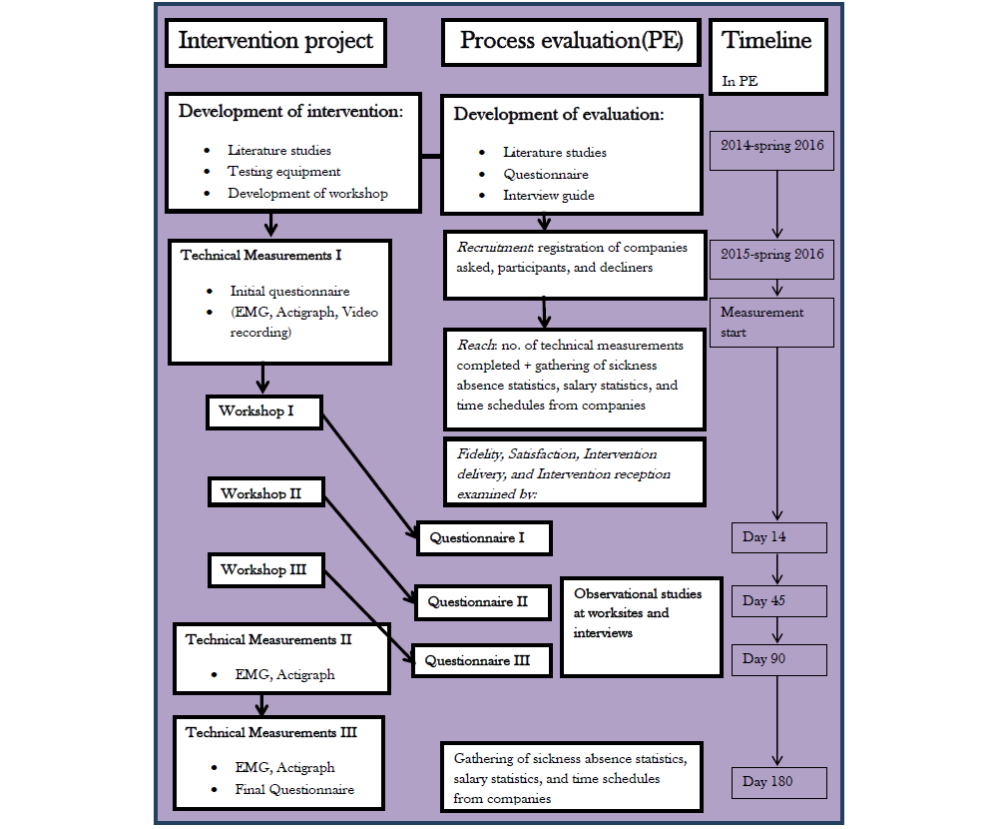
Process evaluation activities and timeline for a participatory intervention to reduce physical risk factors in construction work. EMG: electromyography.

The evaluation is designed in a mixed-methods framework to address different aspects of the intervention, which has been recommended by several sources on evaluation research [30,31]. To assure that our evaluation addresses key pieces of information about the interventions, we draw upon an adapted version of the framework presented by Saunders et al for process evaluation in public health interventions [32-34]. More specifically, using this framework means addressing *recruitment, reach, fidelity, satisfaction, intervention delivery*, *intervention received*, and *context* [34]. Because previous studies have used these terms differently, we define their specific use in the present study below. Figure 1 shows the timeline for the intervention project and process evaluation. Textbox 1 shows the description of evaluation components.

**Process evaluation components and description of their elements.**

Recruitment

- Number of companies asked to participate
- Number of companies agreeing to participate
- Number of gangs participating (intervention and control groups)
- Number of workers participating (intervention and control groups)
- Number of workers responding to questionnaires
- Number of workers asked to participate in interviews
- Number of workers participating in interviews
- Number of occupational health and safety (OHS) staff asked to participate in interviews
- Number of OHS staff participating in interviews

Reach

- Number of technical measurements completed
- Number of workshops completed

Fidelity

- The extent to which the workshop program was completed in accordance with intentions

Satisfaction

- Workers’ and OHS staff’s satisfaction with prioritized risk factors, the competencies of workshop facilitators, and the time for workshops
- Workers’ and OHS staff’s satisfaction with the method of the workshops
- Workers’ and OHS staff’s satisfaction with the implementation of measures for improving ergonomic work environment

Intervention delivery

- Perceived intervention implementation according to researchers
- Intervention reception
- Perceived intervention implementation according to workers and OHS staff

#### Recruitment

In our evaluation, recruitment is defined by several parameters: 1) the number of companies asked to participate, and 2) the number of companies agreeing to participate. We will also record 3) the number of work gangs randomly assigned to the intervention and control groups, as well as how many of these completed the study, 4) the number of workers and OHS staff asked to participate in the intervention at each participating company, to answer questionnaires and to participate in interviews, and 5) the number of workers and OHS staff agreeing to participate in the study, to answer questionnaires and to participate in interviews.

#### Reach

Reach can be defined as the proportion of the intended recruited participants who actually received the intervention, as defined by Saunders et al [34]. In our study, the reach of the intervention is defined as the number of technical measurements and workshops completed in each gang of the project in total. We will list the number of workshops that we deliver and will note the reasons for cancellation. We will also list the number of participants volunteering to take part in technical quantitative measurements and workshops, will note the reasons for individual absence.

#### Fidelity and Satisfaction

After each workshop, we will ask all participants to complete a questionnaire addressing workers’ and OHS staff’s perceptions of the workshop. Fidelity will be addressed in 2 ways. First, we will ask workers to rate each workshop phase on a 5-point scale (very bad, bad, neutral, good, very good). Second, we will record all activities of the workshops to assess whether they were completed in accordance with the planned activities.

Likewise, we will assess satisfaction based on the workers’ and OHS staff’s satisfaction with 1) prioritized risk factors, 2) the competencies of workshop facilitators, 3) the time frames for the workshops, 4) the structure of the workshops, and 5) the implementation of solutions for lowering physical exertion and improving the work environment. All of these parameters will be rated on 5-point scales (very unsatisfied, unsatisfied, neutral, satisfied, very satisfied). Through qualitative interviews in selected cases, we will gain further knowledge of workers’ and OHS staff’s satisfaction with and knowledge of particularly well-functioning or dysfunctional elements of the intervention. This will allow us to use the evaluation in a formative manner, adjusting the intervention for better implementation, as previously suggested [35,36].

#### Intervention Delivery

What we term *intervention*, *delivery*, and *reception* is based on the definition by Saunders et al [34], originally termed *dose delivered*. Referring to a complex organizational and ergonomic intervention as a dose does, however, in our opinion, risk confusing the intervention with a medical injection, which would be misleading because the intervention is a framework for developing preventive solutions that intra-acts *with* other agencies in work and organizations. The intervention should not be seen as something we inject into the organization with determinate effects.

Immediately after completing each day of technical measurements and each workshop, the facilitating researchers will have an evaluative meeting. Minutes of the meeting will be produced to evaluate the success of the activities. Furthermore, we will conduct observational studies at the worksites during 2–5 workdays between workshops I and III in order to assess whether solutions on how to reduce excessive workloads were actually used during work. We also record the number of visits and observations during these visits.

#### Intervention Received

After each workshop, we will evaluate the intervention received by using questionnaires. We will ask each of the workers and OHS staff to assess whether the decided measures have been implemented and integrated as part of work. This will be answered on a 5-point scale (very low degree, low degree, moderate degree, high degree, very high degree). More precise descriptions of the measures that were actually implemented will be explored through interviews with participants.

#### Context

We will investigate the intervention context through interviews with workers and OHS staff to explore their positioning in relation to different agencies intra-acting with the intervention. To address the subjective, social, and organizational practices intra-acting with the implementation of the project, we have designed an in-depth qualitative study consisting of observations, interviews, and document analysis. The qualitative design focused on a limited number of intervention cases offering insights on productivity, sickness absence, and time frames, as well as how the intervention intra-acts with worker identity and meaning in work as initially described.

For the qualitative study we will select 4 work gangs based on a *critical case* argumentation for validity and generalization from case study research. Our aim is to select 2 cases (gangs) in which the implementation seems to be particularly successful, and 2 cases in which the intervention seems to meet resistance or other barriers. By selecting “best” and “worst” cases, we have the opportunity of making generalizations of the type “if the implementation meets X as a barrier/facilitator in this case, it will be likely/unlikely to work better/worse in other cases’ [37].

### Initial and Final Questionnaires

We will distribute the survey questionnaires to the workers of both the intervention and control groups at the time of the initial technical measurements and again after the intervention period. The questionnaire will contain questions addressing the physical work environment and several risk factors of particular relevance to work in the construction industry drawn from previous research [3,22,38]. Of particular interest are questions related to the worker’s capacity for taking care of the body in work, their physical exertion, managerial support for improving health and safety at work, the worker’s influence on health and safety, and the availability and usage of technical assistive devices.

### Questionnaires I, II, and III

These questionnaires will be handed out to participants of the intervention group directly after each workshop and will address fidelity, satisfaction, and the intervention delivered.

### Interviews

We will conduct interviews with the intervention group and address the workers’ and OHS staff’s satisfaction and context. As described for *context* above, we will select cases for interviews through a critical case definition, as discussed by Flyvbjerg [37]. Based on this, we will select cases in which the intervention seems to be working particularly well, and cases where the intervention meets particular complications (best and worst cases). From this selection strategy, we will be able to make generalizations of the sort “if this is (not) valid for this case, then it applies to all (no) cases” (p. 230 in [37]). The interviews are aimed at producing knowledge about how the participants make sense of the intervention. To gain in-depth insight into the practices taking place in relation to the implementation, we will interview workers, health and safety professionals, and managers engaged with the project in each of the 4 cases. We will design and conduct the interviews in a semistructured interview format, where most questions will be posed rather openly and important themes will be explored through follow-up questions, asking the participants to discuss and elaborate their answers. To facilitate discussion and positioning statements among workers, we chose to implement a focus group interview structure, because group negotiations of what is going on at the workplace are an important source of information in the analysis of the scope for changing or developing the working environment. This can be challenging with regard to managers and OHS staff, as these groups are often singularly represented on site, but we will carry out focus groups where possible. If this proves impossible, we will conduct interviews with managers and OHS staff individually.

We will conduct the interviews between workshops II and III to ensure that the participants have the intervention fresh in their memories, while also having some experience of the action taking place during the intervention.

All interviews will be transcribed and imported to NVivo (QSR International Pty Ltd) for coding and to facilitate the analysis. The interview analysis will draw on a view of lingual productions as positioning in discursive-material practice in an analytical framework composed on the thoughts of Davies and Harré [39] and Barad [40]. Positioning theory in an agential realist framework is a theory conceptualizing the ways in which people (re)configure subjectivities through lingual and physical engagement with other people and the materialities of the world, including work. This approach will allow us to analyze how participants draw on elements of work and the body, as well as social and organizational practices, to describe and rationalize their experiences of the intervention.

### Observations

Observations will be conducted in 2 forms and particularly address the delivery of the intervention as well as the context. First, we will video record all workshops in the project to permit analysis of the processes taking place during the workshops. This analysis will illustrate the communication concerning lowering physical exertion through the intervention. In particular, we are interested in how participants use the technology and what solutions are reachable in the interplay between workers, health and safety professionals, and management. We will also analyze this observational material through positioning theory [39] in a modified agential realist framework drawing on Barad [40], allowing a focus on positioning of both physical and subjective characteristics of work and the intervention.

Second, a researcher will follow the work at the intervention sites during the intervention period in order to gain insight into the practices in the organization during the time of implementation. These observations will be drawing on observational methodology and analysis by Czarniawska, who suggests observing particular practices, objects, or people [41]. From these suggestions, we intend to follow practices and objects of particular relevance to the solutions on how to reduce excessive workloads developed in the intervention. During these observations, we will record field notes for analysis. These notes will be taken immediately after each day of observation elaborated by the conducting researcher and used for analysis of practices in the organizations and worksites where the interventions are conducted.

### Document Analysis

In the participating companies, we will obtain documentation of sickness absence, time frames, and economic measurements (performance-based salary levels or budget numbers). Through analysis of these documents, we will attempt to gain insight into some of the measures of how the intervention affects productivity, which is very important to estimating the potential costs of this type of intervention.

### Mixed-Methods Analysis

We will analyze the different empirical elements of the study in relation to each other. We will focus on combining triangulation and a complementary approach to the mixed-methods design rather than on quantifying qualitative material or other approaches to mixed methods [42]. In practice, our mixed-methods analyses will be focused on discovering consistencies and inconsistencies in the physical measurements, questionnaires, qualitative data, and document. Further, we will use our qualitative data to elaborate on the physical measurements, which are of limited character. By pursuing this approach, we can analyze in a detailed manner how the intervention functioned in the different settings, in line with Bamberger et al [30]. Table 1 summarizes the tools used for each evaluation component.

**Table 1:** Tools used for each component.

Component	Methodological tool
Recruitment	Checklist, questionnaires, interviews
Reach	Checklist
Fidelity	Summary of researchers’ evaluation discussion
Satisfaction	Questionnaires I, II and III, and interviews
Program delivery	Summary of researchers’ evaluation discussion, observational studies
Program reception	Questionnaires I, II and III, and interviews

## Results

At the time of submitting this paper (February 2016), we have made initial contact with cases for the intervention and evaluation, and 40 workers have agreed to participate so far. We have completed evaluation design and have prepared interview guides and the observational methodology. The intervention and evaluation are designed to run over the course of winter 2015 to winter 2016, after which time we will undertake the analysis.

## Discussion

One practical issue when conducting research, interventions in particular, in the construction industry can be that time frames are often very narrow, which is why workers often do not have the time to participate in interventions. One particular challenge will be to engage workers, OHS staff, and managers to cooperate in developing and implementing solutions for reducing physical exertion in work, as these groups are usually not engaged in such activities. These challenges can complicate our aim to complete the workshops and group interviews. We will, however, attempt to follow through.

A particular strength of the evaluation design is the mixed-methods framework. Through using lingual communications, observed practices, and quantified assessments, we have configured an analytical apparatus capable of describing how the intervention intra-acts with the workplaces in which it is implemented. We get the opportunity to identify mutually supportive configurations of these relationships, as well as the ability to identify differences occurring through only one or two methodological approaches.

Also, the formative evaluation design allows us to use the insights produced through the evaluation to customize parts of the intervention to better address the context’s or participants’ requirements for a fruitful implementation.

However, we do expect that completing the mixed-methods analysis of the various sources of data and measurements will pose significant challenges. For instance, weighing the different approaches in relation to each other will demand attention and carefulness in the research group working with the study. For these reasons, we expect to be able to illustrate how agencies in construction work intra-act in relation with the ergonomic intervention and to reconfigure the boundaries of material work, organization of physically exerting work tasks, and worker subjectivity. These insights will provide pointers for future action and interventions targeting physical exposure in construction work.

## Abbreviations

OHS: occupational health and safety
